# Experimental and Numerical Investigation on the Size Effect of Ultrahigh-Performance Fibre-Reinforced Concrete (UHFRC)

**DOI:** 10.3390/ma14195714

**Published:** 2021-09-30

**Authors:** Andreas Lampropoulos, Demetris Nicolaides, Spyridon Paschalis, Ourania Tsioulou

**Affiliations:** 1 School of Architecture, Technology and Engineering, University of Brighton, Cockcroft Building, Lewes Road, Brighton BN2 4GJ, UK; o.tsioulou@brighton.ac.uk; 2 School of Engineering, Department of Civil Engineering, Frederick University, 1036 Nicosia, Cyprus; d.nicolaides@frederick.ac.cy; 3 School of Engineering and Computing, University of West London, Lady Byron Building, St Mary’s Road, London W5 5RF, UK; Spyros.Paschalis@uwl.ac.uk

**Keywords:** UHPFRC, strengthening, flexural strength, size effect, constitutive stress–strain model, numerical modelling

## Abstract

In the last few years, there has been increasing interest in the use of Ultrahigh-Performance Fibre-Reinforced Concrete (UHPFRC) layers or jackets, which have been proved to be quite effective in strengthening applications. However, to facilitate the extensive use of UHPFRC in strengthening applications, reliable numerical models need to be developed. In the case of UHPFRC, it is common practice to perform either direct tensile or flexural tests to determine the UHPFRC tensile stress–strain models. However, the geometry of the specimens used for the material characterization is, in most cases, significantly different to the geometry of the layers used in strengthening applications which are normally of quite small thickness. Therefore, and since the material properties of UHPFRC are highly dependent on the dimensions of the examined specimens, the so called “size effect” needs to be considered for the development of an improved modelling approach. In this study, direct tensile tests have been used and a constitutive model for the tensile behaviour of UHPFRC is proposed, taking into consideration the size of the finite elements. The efficiency and reliability of the proposed approach has been validated using experimental data on prisms with different geometries, tested in flexure and in direct tension.

## 1. Introduction

The majority of the existing Reinforced Concrete (RC) structures need to be upgraded, either because they are designed with old seismic code provisions or without them entirely, or because of existing damages. Nowadays, there is a wide range of techniques for the structural upgrade of existing RC elements, and the use of novel high-performance materials has been shown to offer enhanced structural performance and durability. Ultrahigh-Performance Fibre-Reinforced Concrete (UHPFRC) is a relatively new construction material with superior mechanical characteristics. It is characterised by significantly enhanced compressive and tensile strength, and exceptional ductility and energy absorption capacity. These characteristics are directly linked to the mix design, and there are numerous published studies where the effect of the mix design, the type and size of aggregates, and more significantly the dosage and characteristics of steel fibres have been examined [1,2,3,4,5,6,7,8,9,10,11,12,13,14,15,16,17,18,19,20].

In most cases, the behaviour of UHPFRC is significantly affected by the microstructure of the cementitious matrix and the characteristics of the fibres, since the strain-hardening characteristics are attributed to the bond between the fibres and the matrix, which is directly linked to the bridging effect of the fibres.

The effect of different type, length and volume fraction of fibres on the mechanical properties of UHPFRC has been examined by a number of researchers [5,6,7,8,9,10,11]. Paschalis and Lampropoulos [5] found that an increase in the steel fibre content from 1 to 6 Vol.-% enhanced the tensile strength by 92% and the compressive strength by 72%. Hannawi et al. [6] examined various types of fibres, and for a volume fraction of 1%, they found that the effect of the fibres on the compressive strength and the elastic modulus of UHPFRC specimens was negligible. Abbas et al. [7] explored the effects of the steel fibre length and volume fraction on the mechanical properties and durability of the UHPFRC. Based on this study [7], the addition of steel fibres significantly enhanced the tensile and flexural strength, while the compressive strength was only slightly increased. It was also observed that the addition of fibres altered the failure pattern from sudden and explosive to ductile behaviour. The length of the fibres had negligible effect on the compressive strength, but they considerably affected the peak load carrying capacity and load-deflection behaviour. Gesoglu et al. [8], tested the effect of microsteel, hooked steel and microglass fibres vol % up to 2% on the properties of the UHPFRC. They observed that an increase in fibre content led to increased compressive, tensile and flexural strength and increased modulus of elasticity of the UHPFRC regardless the fibre type. However, the strength values began to decrease after 1.5% volume of glass fibres, while 2% hooked steel fibres led to UHPFRC enhanced ductility. Kazemi et al. [9] examined the mechanical properties of UHPFRC containing up to 5% volume fraction of smooth steel fibres. The key findings were that an increase in the fraction of steel fibres led to significant increases in flexural and shear strength. Wu et al. [10,11] investigated the influence of straight, corrugated, and hooked fibres on fibre–matrix bond properties, and compressive and flexural properties of UHPFRC. It was found that the compressive and ultimate flexural strengths increased with the increase in fibre content and age. Additionally, pull-out bond strength and toughness of embedded hooked fibres were much higher compared with those with straight and corrugated fibres. Yoo et al. [12], conducted four-point bending tests on UHPFRC beams with smooth steel fibres of different length. Based on this study [12], fibre length significantly increased the load and toughness of the beams after the limit of proportionality, due to the improved fibre bridging capacity. In addition, beams with a longer fibre length exhibited a higher number of microcracks.

Another important parameter is the orientation of the fibres, which is affected by the method of pouring and by the dimensions of the examined specimens. There are a few studies on the so-called “size effect” of UHPFRC [9,13,14], which prove that the size of the examined specimens is important for both the compressive and flexural strength characteristics. Kazemi et al. [9] observed that smaller samples tend to show higher compressive and direct shear strength. Mahmud et al. [13] investigated the size effect on the flexural strength of UHPFRC beams tested under three-point bending tests. Results showed that the size effect on the flexural strength of UHPFRC is negligible and follows the yield criterion because of its high ductility. An et al. [14] examined the size effect on the compressive strength of UHPFRC cubes with different sizes, and they found that the larger specimens had lower compressive strength when compared with the smaller ones. Even if the existing studies present some useful experimental data on the performance of UHPFRC specimens with carrying sizes, the so-called “size effect” has not been sufficiently explained and there is not any available methodology on how to take this size effect into consideration for practical applications.

The addition of fibres to the concrete matrix can dramatically improve the overall mechanical performance and fracture behaviour of the composite and can also impart it with additional strength in tension, shear and flexure [5,6,7,8,9,10,11]. It is, however, extremely difficult to achieve an even distribution of fibres within the mix, especially when a large quantity of fibres is being used. Failure to attain this goal may result in low mechanical properties, whereas a proper and even fibre distribution can guarantee considerably higher values of properties. This is more evident in the case of UHPFRC, where (i) the interfacial bond between the fibres and the matrix is particularly strong, due to the dense structure of the material and (ii) unreinforced matrices are extremely brittle due to the absence of coarse aggregates in these types of materials. The mechanical and fracture properties of any UHPFRC depend to a high degree on the uniform distribution of fibres in the bulk of the material. Any regions with a low concentration of fibres, or with no fibres, are potential sites of weakness. The distribution of fibres in the mix depends on a number of factors, such as how the fibres were introduced into the mix, on the vibration frequency during compaction, and on the size and shape of the object cast from UHPFRC [1,2,4]. The difficulty in achieving an even distribution of fibres is more pronounced in thicker specimens (e.g., 100 mm), whereas an even distribution can be achieved without difficulty in specimens with a relatively small thickness (e.g., 5 mm–30 mm) [1,2,3]. This particular observation is important, as it can be a crucial factor towards the discrepancy of the experimental results between specimens of several sizes [1,2,3]. The energy absorbing mechanisms (expressed through the size of the Fracture Process Zone (FPZ)) are reduced in the case of larger UHPFRCC specimens because of the difficulty to achieve a uniform fibre distribution, and thus engage more mechanisms in the energy absorbing process. On the other hand, specimens of smaller thicknesses exhibit an even distribution of fibres which apply substantial closure pressure, thus increasing the flexural capacity of the beams [1,2]. Previous research [1,2,3] that thoroughly examined the failure surfaces of three-point bend-tested beams of several sizes confirmed the aforementioned remark. Awinda et al. [15] performed experimental and numerical investigations on UHPFRC prisms with various geometries, where a sensitivity analysis was conducted. According to this study [15], the fibres’ orientations and the alignment of the fibres seem to be quite prominent for specimens with depths of 50 mm or less. It was also highlighted that further work is required to consider the effect of parameters such as the fibre content and length on the numerical modelling of UHPFRC [15]. This is a particularly important aspect, since the application of UHPFRC elements with small thickness, such as bridge decks and strengthening layers, has been extensively used in the last few years. The use of additional UHPFRC layers or jackets has been shown to be quite effective for the enhancement of the flexural and shear capacity of Reinforced Concrete (RC) structures [17,18,19,20]. Additionally, the application of UHPFRC layers in connection with existing RC slabs has been found to offer significant improvement of the punching shear resistance [21], and the enhancement of the punching shear of the composite/strengthened elements can be calculated using an analytical model [22]. In all these applications, thin UHPFRC are used, however the size effect is not taken into consideration and the tensile characteristics are derived using either prisms tested under flexural loading or from the direct tensile testing of dog-bone shaped specimens with various geometries. The current study aims to examine the effect of steel fibres volume fraction and the effect of the dimensions of the examined specimens on the flexural strength characteristics of UHPFRC, and to propose a suitable methodology for the numerical modelling of UHPFRC elements with various geometries and thicknesses.

## 2. Experimental Investigation

In this paper the results of two different experimental studies have been combined and used to investigate a number of different depths of UHPFRC prisms. Flexural tests have been conducted on prisms with various geometries and these results have been used for the validation of the numerical model. Information related to these experimental works (e.g., materials’ mix designs, manufacturing processes, experimental results, etc.) are presented in the following sections.

### 2.1. Material Preparation and Geometry of the Examined Specimens

In the present paper, two different UHPFRC mix designs (i.e., UHPFRC-1 and UHPFRC-2) have been selected and used. The selection of the particular mixtures was made in order to accumulate a sufficient number of different depths of prisms, thus allowing for investigation on the size effect on the performance of UHPFRC layers. UHPFRC-1 was developed at Cardiff University (Cardiff, UK), as is described by Nicolaides [1], whereas UHPFRC-2 is based on the experimental investigation of Hassan et al. [16]. The mix proportions for the two mixtures are provided in Table 1.

Both mixtures are characterized by the use of high cement content 52.5R, along with the use of microsilica and low water–binder ratios, which were achieved by the use of superplasticizers. The maximum particle sand size for UHPFRC-1 was 0.6 mm, whereas the corresponding value for UHPFRC-2 mix was 0.5 mm. For the development of UHPFRC-2 mix a considerable amount of Ground Granulated Blast Furnace Slag (GGBS) was also utilized. Large volumes of steel fibres were also used in each mixture, i.e., 6% (468 kg/m^3^) and 3% per volume (234 kg/m^3^) for mixtures 1 and 2, respectively. For the development of UHPFRC-1, a combination of shorter (i.e., 6 mm) and longer (i.e., 13 mm) brass coated steel fibres were added, whereas for the development of UHPFRC-2, only one length of fibres (13 mm) was incorporated in the mixture. All fibres had a diameter of 0.16 mm, a tensile strength of 3000 MPa and Modulus of Elasticity 200 GPa.

Both mixtures were produced in the labs by applying dry mixing process, i.e., mixing of the dry materials (sand, silica fume, cement and GGBS) first, before the addition of any liquid material. In UHPFRC-1, the steel fibres were also added into the dry mixture, just before the addition of water and superplasticizer. In contrast, in UHPFRC-2 steel fibres were incorporated in the mix right after the addition of the liquid constituents of the mixture. For the production of both materials, high-shear pan mixers were used.

The moulds of UHPFRC-1 were left in environmental conditions for 24 h, and then the demoulded specimens were placed into a hot curing tank, filled with water controlled at 90 °C. The specimens were left in the tank for 9 days. On the first day the temperature of the curing tank was increased (20–90 °C), and on the ninth day it was decreased (90–20 °C) gradually, in order to prevent thermal shock of the specimens. The hot curing regime was applied in order to minimize the curing period of the material. The same curing procedure was also followed for the UHPFRC-2, and after demoulding the specimens were placed into a hot curing tank at 90 °C and tested after 14 days.

**Figure 1:**
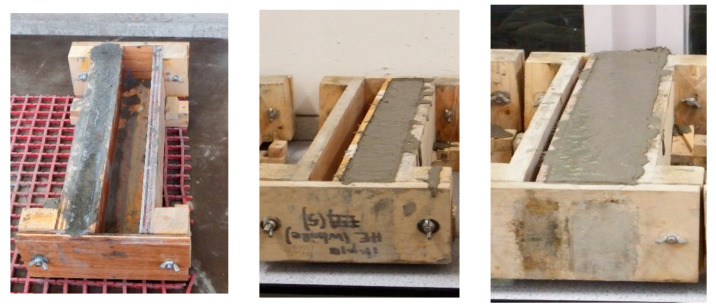
Preparation of prisms with different depths.

From mix UHPFRC-1, two different layer depths were investigated, namely 35 mm and 100 mm, whereas from mix UHPFRC-2, four different layer depths were investigated, namely 25 mm, 50 mm, 75 mm and 100 mm. At least three beams were prepared for each layer depth. In addition, six dog-bone specimens were also prepared and tested in order to determine the direct tensile strength of UHPFRC-2. The corresponding value of the direct tensile strength of UHPFRC-1 was determined in an earlier study by Benson and Karihaloo [23]. In Figure 1, pictures taken during the preparation of beams with different layer depths are presented.

### 2.2. Flexural Prism Tests

**Figure 2:**
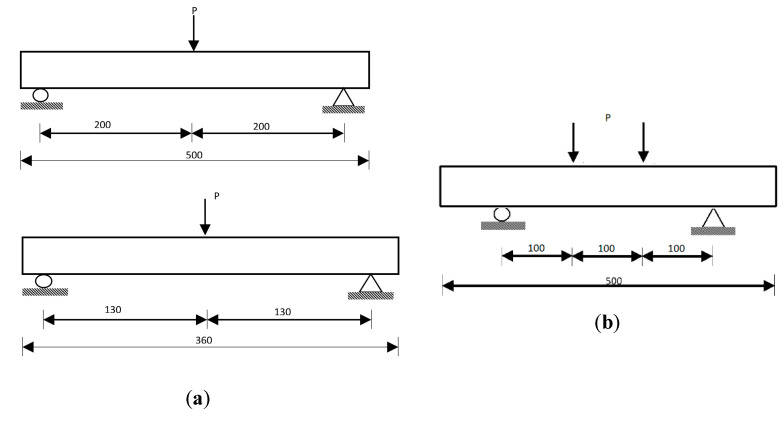
Schematic presentation of (**a**) the three-point bending test (UHPFRC-1) and (**b**) the four-point bending test (UHPFRC-2) (dimensions in mm).

**Figure 3:**
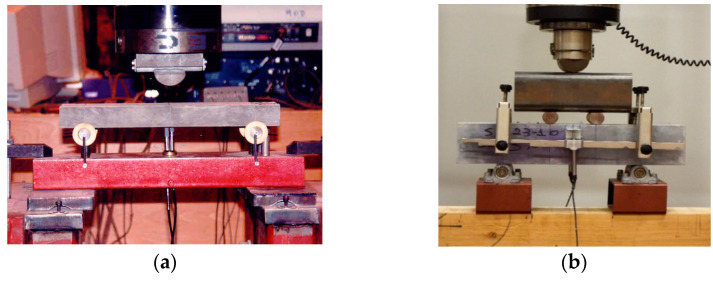
Experimental setup for the testing of (**a**) UHPFRC-1 and (**b**) UHPFRC-2 beams.

In this section, the experimental results of the flexural testing of prisms with different section depths are presented. At least three identical specimens were tested for each different depth. Figure 2 illustrates schematically the experimental setups of (a) the three-point bending test (UHPFRC-1) and (b) the four-point bending test (UHPFRC-2), including the dimensions of both the overall and the testing spans and the location of the applied loads (P). For the determination of the tensile/flexural strength of UHPFRC-1, prisms were tested by three-point bending (Figure 3a) for deformation control. Two types of measurement were recorded for each beam: (1) the load from the load cell of the testing machine; (2) the vertical deflection at the centre point. The vertical deflection was measured by a single LVDT placed underneath the testing beam at the centre point. The tests were performed in a stiff self-straining testing frame. Samples from UHPFRC-2 were tested under four-point loading (Figure 3b). As can be seen in Figure 3b, for the testing of UHPFRC-2, an external yoke was used together with two LVDTs which were attached on both sides of the specimens to record the average deflections of the beams. All tests were conducted under a constant displacement rate of 0.001 mm/sec according to JSCE [24]. The flexural strengths of all specimens were calculated from the recorded data using Equations (2) and (3), depending on the loading conditions.

**Figure 4:**
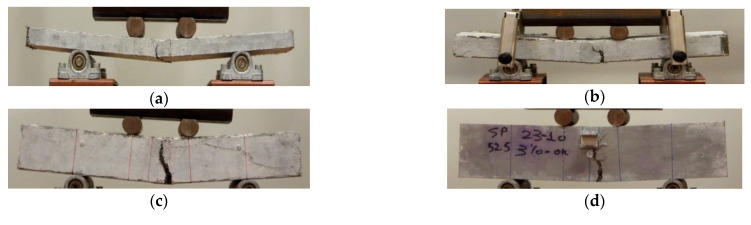
Characteristic failures of selected typical prisms for (**a**) 25 mm, (**b**) 50 mm, (**c**) 75 mm and (**d**) 100 mm depths.

**Figure 5:**
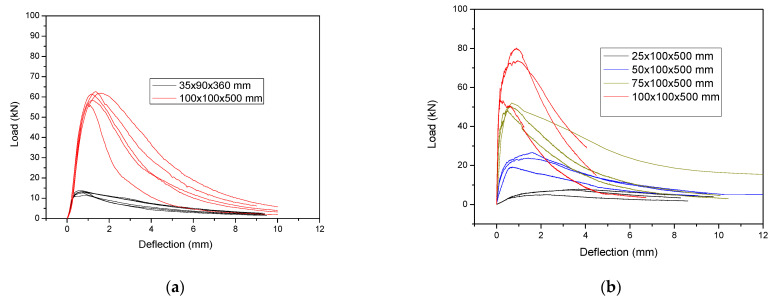
Load-deflection results for all the examined specimens of (**a**) UHPFRC-1 and (**b**) UHPFRC-2.

The typical failure in all the examined cases was formed with a main crack in the middle of the span. The crack patterns for selected typical prisms with different depths are presented in Figure 4. The load-deflection results for all the examined specimens for UHPFRC- 1 and UHPFRC-2 are presented in Figure 5a,b, respectively.

The values of Figure 5 have been used for the calculation of the flexural strength for both UHPFRC-1 and UHPFRC-2.

The flexural strength *σ_t_* is calculated using Equation (1):

(1) $$\sigma_{t}=\frac{M\cdot y\;}{I}$$

where:
*M*is the bending moment;*I*is the moment of inertia;*y*is the distance of the centroid from the extreme fibre.

Using Equation (1), the following models are derived for the three-point Equation (2) and four-point Equation (3) bending testing, respectively, of UHPFRC-1 and UHPFRC-2.

(2) $$\sigma_{t-3p}=\frac{3\cdot P\cdot L\;}{2\cdot b\cdot d^{2}}$$

(3) $$\sigma_{t-4p}=\frac{P\cdot L\;}{b\cdot d^{2}}$$

where:
$\sigma_{t-3p}\;and\;\sigma_{t-4p}$are the flexural strength values calculated from the three-point and four-point bending tests (MPa);Pis the peak load (N);Lis the effective span length (mm);bis the width of specimen (mm);dis the depth of the specimens (mm).

**Figure 6:**
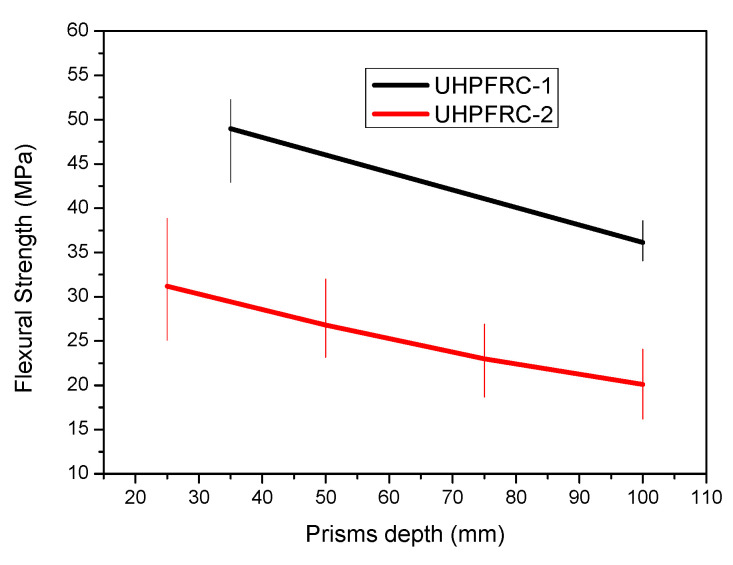
Flexural strength results for UHPFRC-1 and UHPFRC-2 for all the examined prism depths.

The flexural strength results for all the different examined thicknesses have been calculated and the average results together with the scatter plot are presented in Figure 6.

From the results presented in Figure 6, it can be clearly observed that there is a reduction in the flexural strength as the depth of the specimens is increased, which confirms the so called “size effect”. This reduction is attributed to the uneven distribution of fibres in thicker elements (e.g., 100 mm) as opposed to specimens with smaller thicknesses where there is a more even distribution of the fibres and therefore increased flexural strength is achieved. Additionally, the results of Figure 4 show a similar trend in the reduction in the flexural strength values, with a slightly more pronounced rate of reduction in the case of UHPFRC-1 which is linked to the higher percentage of steel fibres (UHPFFRC-1 has 6% steel fibres while UHPFRC-2 has 3%). Additionally, the overall flexural strength of UHPFRC-1 is higher than the respective values of UHPFRC-2 due to the higher percentage of steel fibres.

## 3. Constitutive Modelling and Numerical Analysis

### 3.1. Direct Tensile Tests 

**Figure 7:**
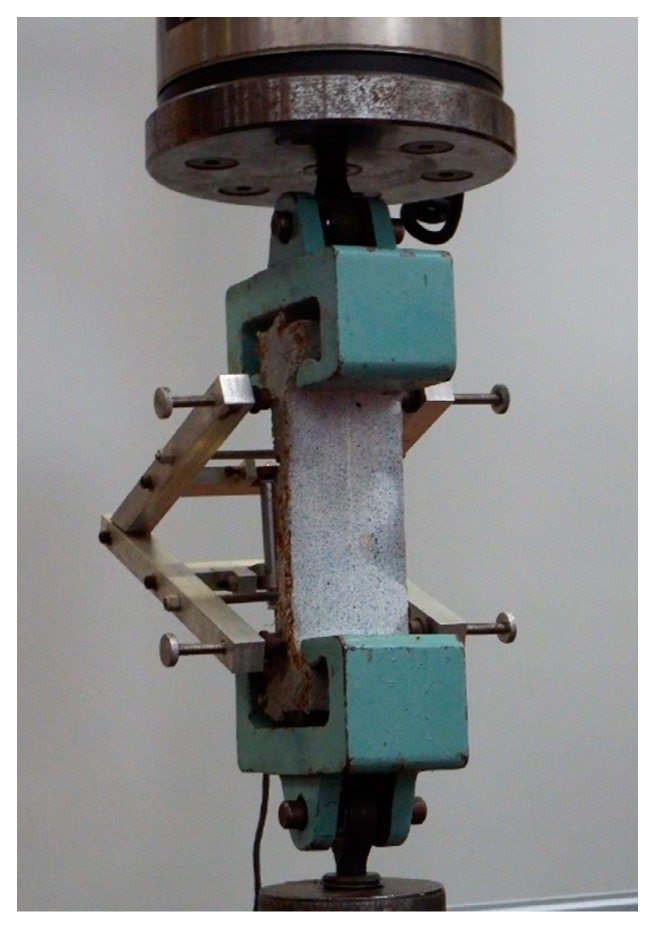
Direct tensile testing of UHPFRC-2 dog-bone specimens.

Direct tensile tests have been conducted for both UHPFRC-1 and UHPFRC-2. The direct tensile test results of UHPFRC-2 have been used for the constitutive modelling of UHPFRC and the values of the model have been accordingly adjusted to consider the different Youngs modulus and tensile strength of the two mixes. In total, six UHPFRC-2 dog-bone specimens were tested under direct tensile testing [17]. A pair of steel grips was used to apply the tensile load and all the specimens were tested under displacement control with a rate equal to 0.007 mm/s. The extension and the respective strain values were calculates using the measurements of Linear Variable Differential Transformers (LVDTs) using the setup presented in Figure 7 [17].

**Figure 8:**
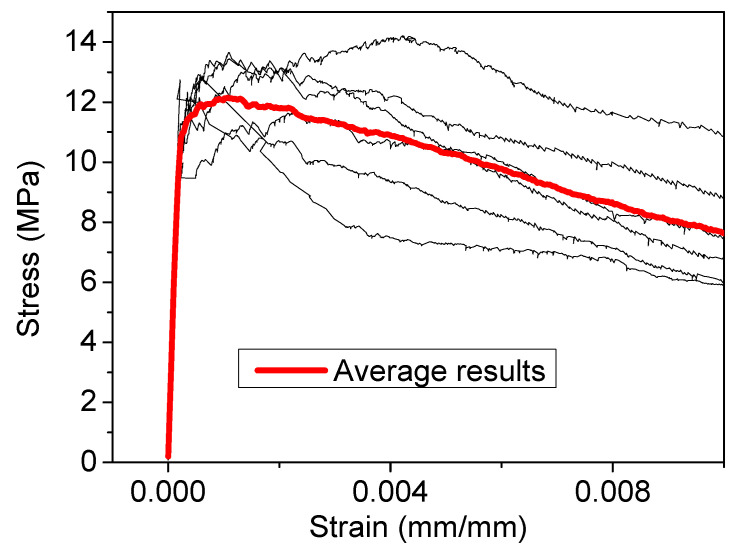
Direct tensile stress (MPa)–strain (mm/mm) results for UHPFRC-2.

The direct tensile test results for the UHPFRC-2 specimens are presented in Figure 8. The results of Figure 8 present the distribution of the stress with the total strain for all the individual specimens together with the average results [17]. According to the direct tensile test results of Figure 8, the tensile strength of UHPFRC-2 was found to be in the range of 11.74 MPa to 14.20 MPa while the respective strength of the average stress–strain curve was calculated equal to 12.15 MPa.

### 3.2. UHPFRC Constitutive Modelling 

**Figure 9:**
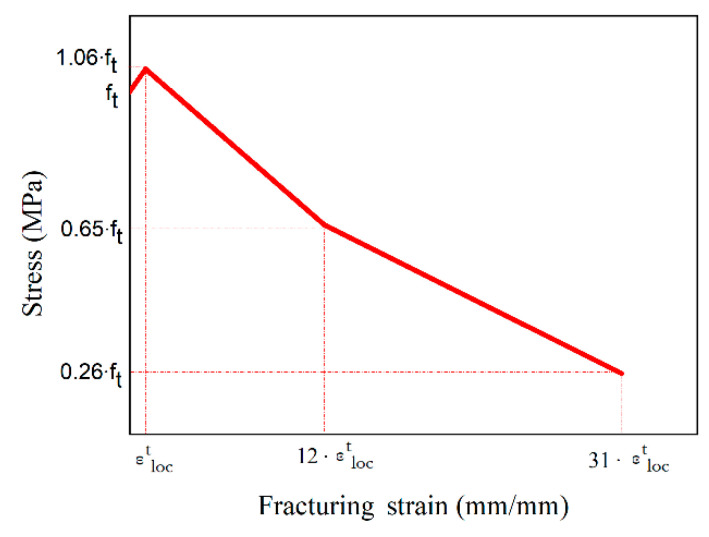
Constitutive modelling for UHPFRC.

The experimental results of Figure 8 were used for the development of the constitutive model presented in Figure 9 which represents the stress–strain distribution after the end of the elastic part. This model consists of a linear part up to the maximum tensile stress value (*f_t_*) followed by a bilinear descending branch (Figure 9). This model will be used for the numerical modelling of both UHPFRC-1 and UHPFRC-2 to evaluate the reliability of the model for these two different types of UHPFRC.

ATENA software was used for the numerical simulations, and experimental values for the tensile strength and the young modulus were used for the modelling of the tensile stress–strain behaviour using the model of Figure 9. For UHPFRC-1, 16 MPa tensile strength was obtained experimentally, while the Youngs Modules and the Compressive strength were equal to 48 GPa and 193.6 Mpa, respectively [1]. Regarding UHPFRC-2, tensile strength of 11.5 Mpa and Youngs modulus equal to 57.5 Gpa were used, while the compressive strength was found equal to 164 MPa.

**Figure 10:**
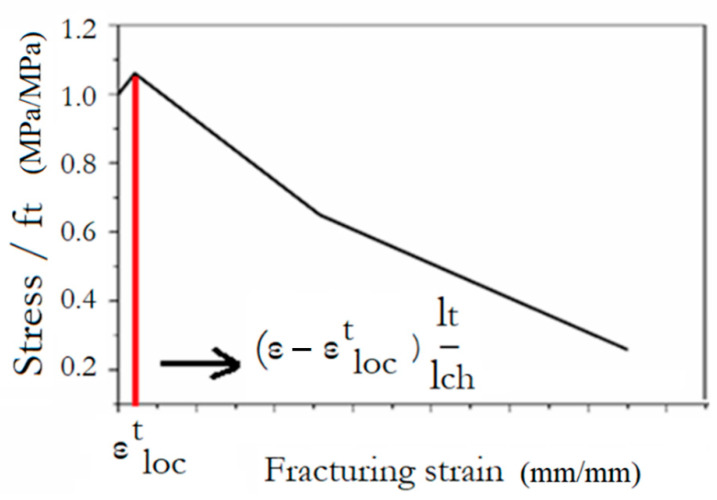
Constitutive modelling for UHPFRC depending on the FEA mesh size.

To determine the strain values for the characteristics points of the model in Figure 9, a strain equal to 0.042 was taken as $\varepsilon_{loc}^{t}$ using characteristic size (*l_ch_*) equal to 2 mm and finite elements size (*l_t_*) equal to 65 mm [17]. This model has been found to be able to accurately predict the behaviour of UHPFRC-2, however the reliability of this model is highly dependent on the values of the characteristic size and the mesh size of the elements of the numerical models, which significantly affect the results in the post-crack region. Therefore, it is important to develop models which can accurately predict the behaviour of UHPFRC independently of the size of the Finite Element Models. This crucial aspect is addressed in this study with the development of a model which takes into consideration the size of the elements and can be used to accurately predict the behaviour of various geometries of UHPFRC specimens. The proposed tensile stress-strain characteristics are defined each time depending on the size of the finite elements (*l_t_*), following the model presented in Figure 10. 

**Figure 11:**
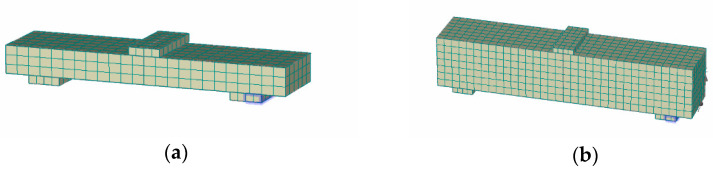
Numerical models for UHPFRC-1 prisms (**a**) 25 × 90 × 360 mm and (**b**) 100 × 100 × 500 mm.

**Figure 12:**
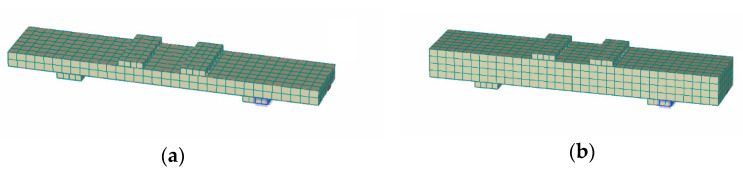

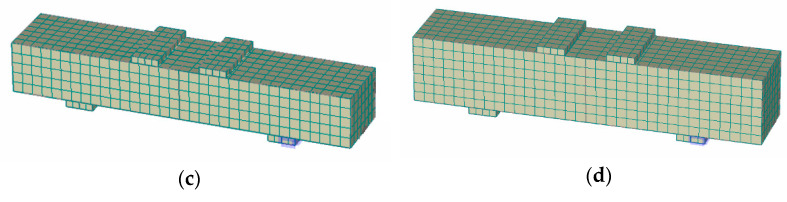
Numerical models for UHPFRC-1 prisms (**a**) 25 × 100 × 500 mm, (**b**) 50 × 100 × 500 mm, (**c**) 75 × 100 × 500 mm, and (**d**) 100 × 100 × 500 mm.

This approach has been examined for both UHPFRC-1 and UHPFRC-2 and the numerical models presented in Figure 11 and Figure 12 have been analysed.

For the specimens of Figure 12b–d, the finite element size (*l_t new_*) was 14.7 mm while the respective value for the specimen of Figure 12a was 15 mm. Following the procedure described in Figure 10, the UHPFRC constitutive model was altered by adjusting the characteristic size value (*l_ch_* = 2 mm) which was initially proposed for *l_t_* = 65 mm, multiplying it with the ratio $\frac{l_{t\;new}}{l_{t}}$ (i.e., for *l_t new_* = 14.7 mm, $\frac{l_{t\;new}}{l_{t}}$ = 0.2 and *l_ch new_* =0.2 ·l*_ch_*=0.4 mm).

Simply supported conditions were applied to all the examined specimens and a monotonically increasing displacement was applied to the middle of the span, reproducing the conditions of the experimental tests. The comparisons between the numerical and analytical results are presented in Section 4.

## 4. Results and Discussion

**Figure 13:**
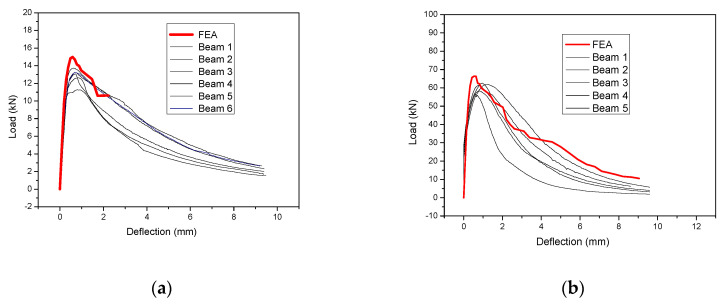
Load deflection results: Numerical vs. Experimental for UHPFRC-1 prisms with (**a**) 25 and (**b**) 100 mm depth values.

**Figure 14:**
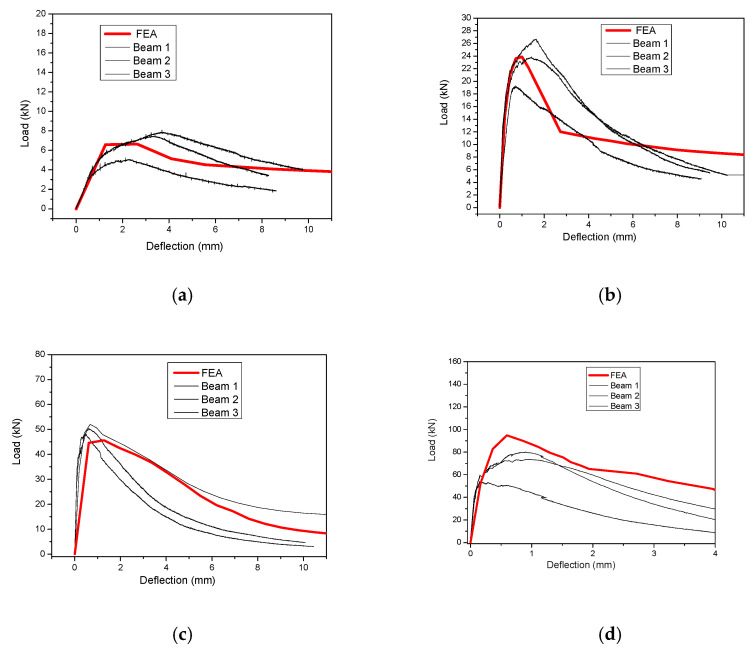
Load deflection results: Numerical vs. Experimental for UHPFRC-2 prisms with (**a**) 25 mm, (**b**) 50 mm, (**c**) 75 mm and (**d**) 100 mm depth values.

The numerical results are compared with the respective experimental results, and the results for UHPFRC-1 are presented in Figure 13 while the respective results for UHPFRC-2 are presented in Figure 14. The results of Figure 13 and Figure 14 show that the numerical modelling results are in a good agreement with all the experimental results for both UHPFRC-1 and UHPFRC-2 for all the examined prism dimensions.

More specifically, the numerical models were found to successfully simulate the initial stiffness, the maximum load and the post-cracking behaviour of most of the examined samples. It should be highlighted here that there is a significant deviation between the experimental results of the multiple samples of each of the examined mixes, and the geometry which is attributed to the distribution of the fibres of the experimental samples. In most of the examined cases the numerical results are approaching the experimental results of the specimens with the highest load capacity (among the numerous specimens examined for each mix/type). Additionally, it is worth mentioning that in the case of specimens with relatively small depth (i.e., 25 mm, 50 mm and 75 mm), the numerical model results are near the average of the experimental results, while in case of specimens with 100 mm the numerical results are slightly higher than the highest values of the experimental results. This is due to the fact that the scatter of the experimental results is linked to the non-uniform distribution and orientation of the fibres, which is more important in the case of specimens with high thickness (i.e., 100 mm). Therefore, the proposed methodology can be used to accurately simulate the response of relatively thin UHPFRC layers and with thicknesses no more than 100 mm. These results prove the applicability of the proposed method for the simulation of UHPFRC specimens with different dimensions, eliminating the impact of the size effect. 

## 5. Conclusions 

In this study, extensive experimental work on prisms with different fibre volume fractions and different geometries has been conducted, and a methodology for the numerical modelling of UHPFRC has been proposed. More specifically, the development of a widely applicably model is proposed for the simulation of UHPFRC independently of the size of the Finite Element Models. The proposed model takes into consideration the size of the elements and can be used to accurately predict the behaviour of various geometries of UHPFRC specimens.

The following conclusions can be drawn:

- The flexural strength of the examined prisms is reduced as the depth of the specimens is increased, which confirms the so called “size effect”.
- This reduction is attributed to the uneven distribution of fibres in thicker elements (e.g., 100 mm), as opposed to specimens with smaller thickness where there is a more even distribution of the fibres and therefore increased flexural strength is achieved.
- The reduction rate of the flexural strength values is more pronounced in case of UHPFRC-1, which is linked to its higher percentage of steel fibres (UHPFFRC-1 has 6% steel fibres while UHPFRC-2 has 3%).
- The proposed numerical modelling approach can accurately predict all the examined types (with different fibre volume fractions and different geometries), confirming the applicability of the proposed method for the simulation of UHPFRC specimens with different dimensions, eliminating the impact of the size effect. The proposed methodology can be used to accurately simulate the response of relatively thin UHPFRC layers and with thicknesses no more than 100 mm. Further research is required for the simulation of UHPFRC specimens with thickness higher than 100 mm.

## Figures and Tables

**Table 1:** UHPFRC 1 and 2 mix designs.

Material	Mix Proportions (kg/m^3^)	
	UHPFRC-1	UHPFRC-2
Cement (52.5N)	855	657
GGBS		418
Silica Fume	214	119
Silica Sand	940	1051
Superplasticizers	28	59
Water	188	185
Steel Fibres	468	234

## Data Availability

Data is contained within the article.
